# Early Detection of Krukenberg Tumors Utilizing ctDNA Testing and CEA Monitoring

**DOI:** 10.1155/crom/5335858

**Published:** 2025-02-21

**Authors:** Angela M. DeRidder, Corrine M. Check, Paul R. Kunk, Christina Martin

**Affiliations:** ^1^Department of Hematology and Oncology, Riverside Health System, Williamsburg, Virginia, USA; ^2^Department of Hematology and Oncology, University of Virginia, Charlottesville, Virginia, USA

## Abstract

Krukenberg tumors are rare cancers involving metastatic disease in the ovaries but classically originate from gastrointestinal malignancies, and often present diagnostic challenges due to their nonspecific symptoms and advanced stage at detection. Traditional imaging techniques like ultrasound, CT, and MRI are common methods of cancer monitoring but are limited in detecting micrometastatic disease and early-stage metastases. Circulating tumor DNA (ctDNA) testing, a noninvasive liquid biopsy method, offers a promising alternative to traditional screening methods, enabling earlier detection and precise molecular profiling of metastatic tumors. We present a case study involving a female patient who initially presented with stage IV colon cancer with oligometastatic disease to a single mesenteric lymph node. Despite neoadjuvant chemotherapy and resection of known disease, postresection ctDNA returned positive. Imaging after metastectomy failed to reveal any sites of ongoing disease, although did show a small, 2.4-cm hypodensity in the right ovary interpreted by radiology as likely an ovarian follicle. Given her ctDNA positivity, she was started on capecitabine. ctDNA levels improved, but her serum carcinoembryonic antigen (CEA) tumor marker continued to rise, and imaging subsequently revealed increased bilateral ovarian masses. She underwent bilateral salpingo-oophorectomy and total abdominal hysterectomy, with pathology confirming metastatic colon adenocarcinoma, and subsequent normalization of her CEA and ctDNA levels. Our findings underscore ctDNA's potential to complement imaging, particularly for high-risk patients, for disease monitoring and to refine therapeutic management when treating Krukenberg tumors.

## 1. Introduction

Metastatic involvement of the ovaries in colon cancer is remarkably rare, occurring with an incidence of approximately 0.8%–7.4% [1, 2]. Known as Krukenberg tumors, these tumors are highly aggressive, often chemorefractory, and typically connotate a poor prognosis. The reported median survival is between 12 and 18 months [3–6], and the 5-year survival rate is about 12%–27% [1, 7–11]. There is currently no standard of care for colon cancer with ovarian involvement given its rareness. However, data suggest that complete resection of the involved ovaries could significantly prolong patients' survival [12, 13]. Unfortunately, due to vague symptomatology and rapid growth, the majority of patients with Krukenberg tumors present with advanced disease, precluding them as surgical candidates with curative intent.

Circulating tumor DNA (ctDNA) is single- or double-stranded DNA released by the tumor cells into the blood [14], and has many potent potential applications in clinical practice, including cancer genotyping and monitoring disease progression or recurrence [15–19]. The use of ctDNA for monitoring for the presence of minimal residual disease after surgery is particularly appealing, as it may allow clinicians to identify relapse earlier than imaging. However, the exact role of ctDNA in cancer care remains to be determined and is currently the topic of a number of clinical trials.

Here, we report a case of ovarian metastases from colon cancer that was identified early using a combination of ctDNA, tumor markers, and imaging, which subsequently enabled the patient to proceed with potentially curative metastasectomy in an otherwise highly fatal disease. This case highlights the potential role of ctDNA testing in detecting and managing Krukenberg tumors.

## 2. Case Report

This case involves a 34-year-old female with no significant past medical history who was diagnosed with iron deficiency anemia 30 weeks into her pregnancy. She received five doses of IV iron, which resolved her iron deficiency, and subsequently delivered a healthy baby at 39 weeks' gestation. Four weeks after delivery, she presented to the emergency department for abdominal pain, fever, nausea, and vomiting. Imaging revealed a large, 6-cm mass in the transverse colon causing obstruction and perforation. The patient went for emergent laparotomy and right hemicolectomy. Pathology revealed moderately differentiated adenocarcinoma of the colon, Grade 2, measuring 6 cm, extending to the mesenteric margin, with 6 out of 28 lymph nodes positive for metastatic adenocarcinoma, final staging pT4aN2bM0. The patient did not have evidence of mismatch repair (MMR) deficiency and as such was not a candidate for immunotherapy. FoundationOne next-generation sequencing showed that her PD-L1 tumor proportion score was 0%, tumor mutation burden was low, and there were no reportable alterations in BRAF, ERBB2, KRAS, or NRAS.

**Figure 1:**
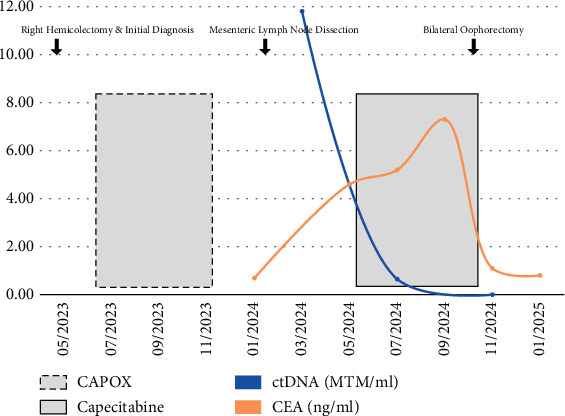
Timeline depicting the course of serum CEA levels and ctDNA levels during treatment, starting at time of diagnosis and the initial right hemicolectomy.

PET/CT was performed 3 weeks postoperatively and revealed a 2.7-cm mesenteric lymph node suspicious for residual metastatic disease. She underwent seven cycles of capecitabine and oxaliplatin (CAPOX). Posttreatment PET/CT showed a decrease in size of the previously demonstrated mass with no associated pathologic fluorodeoxyglucose (FDG) activity, and no additional findings suggesting disease progression or new sites of disease. Carcinoembryonic antigen (CEA) serum tumor marker was not checked prior to surgery or neoadjuvant chemotherapy, but after completion of chemotherapy was normal at < 1.7 ng/mL (see Figure 1).

The patient underwent metastasectomy, with final pathology revealing a 2.7-cm mesenteric lymph node involved with metastatic colonic adenocarcinoma. ctDNA analysis 4 weeks postoperatively returned positive at 11.81 mean tumor molecules (MTM)/mL. CT scans of the chest, abdomen, and pelvis showed no evidence of recurrent or metastatic disease, although it did reveal a benign-appearing, 2.4-cm hypodensity in the right ovary interpreted by radiology as likely representing an ovarian follicle. Repeat CEA at that time remained normal. PET/CT was denied by her insurance and was not pursued further due to financial toxicity.

After significant discussion, given the positive ctDNA findings, she was started on adjuvant capecitabine 2 months after her resection. Repeat ctDNA after 3 months of chemotherapy improved to 0.65 MTM/mL. However, CEA levels during this period rose persistently, reaching as high as 8.2 ng/mL. Repeat imaging with CT of the abdomen and pelvis showed a new, large cystic lesion in the left adnexa, likely arising from the left ovary. Ultrasound was recommended and revealed bilateral ovarian enlargement with multiple simple cysts, read as most likely to be benign in origin. However, given her history of colon cancer, a PET/CT was performed, which showed bilateral enlargement of the ovaries with cystic lesions with intense FDG avidity. MRI of the pelvis confirmed enlarged ovaries with concern for possible primary ovarian cancer versus colonic metastases. She was referred to a gynecologic oncologist, who recommended bilateral salpingo-oophorectomy (BSO) and total abdominal hysterectomy (TAH) due to concern for metastatic disease to the ovaries.

The patient underwent BSO and TAH without complications. Final pathology revealed bilateral ovarian involvement by metastatic adenocarcinoma of colorectal origin. The uterus and biopsies of the peritoneum and the left and right pericolic gutters were negative for additional disease. Two days postoperatively, her CEA decreased to 4 ng/mL, and 2 weeks postoperatively was normal at < 1.7 ng/mL. Repeat ctDNA 3 weeks postoperatively was 0 MTM/mL. At this time, the patient remains in clinical and molecular remission.

## 3. Discussion

Metastatic involvement of the ovaries in colon cancer is remarkably rare, with a reported incidence ranging between 0.8% and 7.4% [1, 2]. Most patients at the time of diagnosis are found to have advanced disease involving not only the ovaries but also the peritoneum and visceral organs [2]. The majority of these cases have been found to be highly chemorefractory, leading to the thought that the ovaries are a “sanctuary for metastases” [20]. Because of low chemotherapy response rates and the high incidence of advanced disease, prognosis for ovarian metastases in colorectal cancer has historically been considered poor [1, 3–11].

More recently, studies have evaluated a small subset of patients with oligometastatic disease and found that resection of ovarian metastases can significantly improve overall survival. One study, performed by Li et al., showed that complete resection of ovarian metastases could improve overall survival by over 10 months compared to palliative resection or no resection at all [12]. Another study, performed by Kagawa et al., showed that the 3-year overall survival rates after curative resection, noncurative resection, and nonresection were 66%, 32%, and 6%, respectively [13]. These studies highlight that the extent of metastasis is a critical factor in determining a patient's overall survival, and that reducing tumor burden by surgical resection may enhance the effectiveness of subsequent systemic therapies, potentially leading to better outcomes.

In this case, we identified a young woman with stage IV colon cancer with oligometastatic disease in a mesenteric lymph node, who was theoretically in clinical remission after curative metastasectomy. However, because of ctDNA positivity, she was started on additional adjuvant chemotherapy. Interestingly, her CEA continued to rise despite improvement in her ctDNA. We believe that at some point after her mesenteric lymph node metastasectomy, micrometastatic disease developed in the ovaries. Our patient was subsequently started on chemotherapy, but because her disease was located in a “sanctuary” site, the disease in her ovaries remained unaffected by chemotherapy and continued to grow, accounting for her rising CEA. Her ovaries continued to shed circulating tumor cells into the blood system, thus accounting for her ongoing ctDNA positivity; however, once freed from the “sanctuary” site of the ovaries, circulating cancer cells were susceptible to chemotherapy again, thereby explaining why her ctDNA levels improved while on capecitabine. Similarly, several other reports on Krukenberg tumors have found disproportionate growth of ovarian metastases while on chemotherapy, with Krukenberg tumors showing continued disease growth despite other sites of concurrent metastases showing chemotherapy response [21, 22]. We believe that our patient's Krukenberg tumors did not respond to chemotherapy because of the ovary being a “sanctuary” site, but the presence of ongoing chemotherapy eliminated circulating cancer cells, allowing her disease to remain confined to the ovaries. Thus, her disease remained confined, and bilateral oophorectomy was therefore potentially curative.

Our case supports the ongoing literature suggesting that early detection of Krukenberg tumors may lead to better outcomes. However, early detection of Krukenberg tumors using traditional surveillance methods can be difficult. Imaging has historically been the gold-standard method for monitoring disease progression and recurrence in patients with advanced colon cancer [23]. In the case of ovarian colonic metastases, however, imaging may be less reliable, as these lesions can be solid, mixed solid/cystic, or predominantly cystic, mimicking simple ovarian cysts. Differentiating between benign ovarian findings and worrisome metastatic lesions can be difficult, particularly in the case of early disease [24]. This was seen in our patient, who had repeat imaging performed 1 month after her metastasectomy, revealing a small, benign-appearing hypodensity in her right ovary, read by radiology as an ovarian follicle. Follow-up imaging with a repeat CT of the abdomen/pelvis and then subsequently pelvic ultrasound continued to report benign-appearing cysts, and it was only when PET/CT revealed abnormal FDG avidity in the ovaries that concern for possible malignancy was raised. Our case highlights how in a young, ovulating patient, traditional imaging methods may be unable to differentiate between normal ovarian follicles, benign ovarian cysts, and malignant disease, especially in the setting of limited disease.

Serum tumor markers are often used in conjunction with imaging for disease monitoring. Serum CEA, an oncofetal glycoprotein that is normally expressed by mucosal cells and often overexpressed various malignancies, can sometimes be helpful in monitoring for colon cancer recurrence or progression. However, these results can sometimes be misleading, as CEA levels can be elevated in both malignant and benign etiologies, including ovarian cysts [25]. One case series showed that benign dermoid cysts can be associated with elevated CEA levels as high as 7.2 ng/mL, with elevated levels correlating with larger cysts. In our patient, CEA levels were initially normal after her metastasectomy but rose concurrently with the development of her ovarian lesions. Had we relied solely on her CEA and imaging as predictors of disease recurrence, and therefore initiation of treatment, therapeutic management could have been significantly delayed as her imaging was initially read as benign, and her initially mildly elevated CEA levels could have been attributed to her ovarian cysts.

ctDNA analysis has many potential applications in clinical practice, including cancer genotyping and monitoring disease progression or recurrence [15–19]. The use of ctDNA for monitoring for the presence of minimal residual disease after surgery is particularly appealing, as it would allow clinicians to identify relapse earlier than imaging. The GALAXY study, performed by Kotani et al., found that among 1039 colon cancer patients with stage II/III disease, postsurgical ctDNA positivity (at 4 weeks after surgery) was associated with higher recurrence risk (hazard ratio (HR) = 10, *p* < 0.0001) and was the most significant prognostic factor associated with recurrent risk (HR: 10.82, *p* < 0.001) [26]. Furthermore, patients with postsurgical ctDNA positivity derived greater benefit from adjuvant chemotherapy compared to patients with negative postsurgical ctDNA (HR = 6.59, *p* < 0.0001). The BESPOKE CRC study had similar findings. Among 295 stage II/III colon cancer patients who underwent curative surgical resection, minimal residual disease positivity was significantly associated with inferior disease-free survival (HR = 20.8, 95% confidence interval (CI): 10.0–43.4, *p* < 0.0001) [27]. Furthermore, among patients with postsurgical minimal residual disease positivity, administration of adjuvant chemotherapy significantly improved disease-free survival (18.7 vs. 6.7 months; HR = 3.9, 95% CI: 1.3–11.5, *p* = 0.01). These studies suggest that ctDNA may have both a prognostic and predictive role in colon cancer management in the future. Currently, however, the National Comprehensive Cancer Network (NCCN) guidelines state that there is insufficient data to recommend the use of postsurgical ctDNA to estimate the risk of recurrence or determine adjuvant therapy [28]. Following the NCCN guidelines, given her negative imaging and tumor marker workup, no additional systemic chemotherapy was warranted for our patient. However, an in-depth conversation was held with the patient regarding the risk of recurrence and the lack of data regarding the predictive value of ctDNA in terms of potential chemotherapy benefit. After careful consideration, given her young age, otherwise excellent health, and her elevated risk of disease recurrence based on the studies cited above, she agreed to start single-agent capecitabine. More aggressive systemic therapy was considered; however, given the lack of predictive data regarding the role of ctDNA and adjuvant chemotherapy, we ultimately felt that the risks outweighed the benefits in the setting of otherwise undetectable disease.

In our patient, use of ctDNA allowed us to identify early signs of disease relapse after our patient's theoretically curative metastasectomy and initiate a conversation regarding the role of additional chemotherapy. However, our case highlights that ctDNA must be used in conjunction with traditional monitoring techniques, such as imaging and serum cancer markers. ctDNA can only reveal tumor DNA in the blood system, and in patients who have disease with limited cancer cell shedding or, in our case, disease in sanctuary sites such as the ovaries, ctDNA response may not reflect actual tumor progression or regression.

## 4. Conclusion

This case adds to the small body of literature describing oligometastatic colonic Krukenberg tumors and is one of the first to describe the potential role of ctDNA in identifying these tumors prior to the development of additional metastatic disease, thus allowing for potentially curative surgical resection in an otherwise highly fatal disease with high morbidity. ctDNA analysis should be used in conjunction with traditional cancer monitoring tools, such as imaging and serum tumor marker testing, for therapeutic management.

## Data Availability

The data that support the findings of this study are available on request from the corresponding author. The data are not publicly available due to privacy or ethical restrictions.

## References

[1] Hanna N. N., Cohen A. M. (2004). Ovarian neoplasms in patients with colorectal cancer: understanding the role of prophylactic oophorectomy. *Clinical Colorectal Cancer*.

[2] Van der Meer R., de Hingh I. H. J. T., Bloemen J. G., Janssen L., Roumen R. M. H. (2022). Role Of Ovarian Metastases In Colorectal Cancer (ROMIC): a Dutch study protocol to evaluate the effect of prophylactic salpingo-oophorectomy in postmenopausal women. *BMC Women's Health*.

[3] Taylor A. E., Nicolson V. M. C., Cunningham D. (1995). Ovarian metastases from primary gastrointestinal malignancies: the Royal Marsden Hospital experience and implications for adjuvant treatment. *British Journal of Cancer*.

[4] O'Brien P. H., Newton B. B., Metcalf J. S., Rittenbury M. S. (1981). Oophorectomy in women with carcinoma of the colon and rectum. *Surgery, Gynecology & Obstetrics*.

[5] Perdomo J. A., Hizuta A., Iwagaki H. (1994). Ovarian metastasis in patients with colorectal carcinoma. *Acta Medica Okayama*.

[6] Graffner H. O., Alm P. O. A., Oscarson J. E. A. (1983). Prophylactic oophorectomy in colorectal carcinoma. *American Journal of Surgery*.

[7] Tan K. L., Tan W. S., Lim J. F., Eu K. W. (2010). Krukenberg tumors of colorectal origin: a dismal outcome—experience of a tertiary center. *International Journal of Colorectal Disease*.

[8] Kim D. D., Park I. J., Kim H. C., Yu C. S., Kim J. C. (2009). Ovarian metastases from colorectal cancer: a clinicopathological analysis of 103 patients. *Colorectal Disease*.

[9] Bakkers C., van der Meer R., Roumen R. M. (2020). Incidence, risk factors, treatment, and survival of ovarian metastases of colorectal origin: a Dutch population-based study. *International Journal of Colorectal Disease*.

[10] Mori Y., Nyuya A., Yasui K. (2018). Clinical outcomes of women with ovarian metastases of colorectal cancer treated with oophorectomy with respect to their somatic mutation profiles. *Oncotarget*.

[11] Jiang R., Tang J., Cheng X., Zang R. Y. (2009). Surgical treatment for patients with different origins of Krukenberg tumors: outcomes and prognostic factors. *European Journal of Surgical Oncology*.

[12] Li X., Huang H., Ran L. (2020). Impact of ovarian metastatectomy on survival outcome of colorectal cancer patients with ovarian metastasis: a retrospective study. *Cancer Management and Research*.

[13] Kagawa H., Kinugasa Y., Yamaguchi T. (2024). Impact of resection for ovarian metastases from colorectal cancer and clinicopathologic analysis: a multicenter retrospective study in Japan. *Annals of Gastroenterological Surgery*.

[14] Sorenson G. D., Pribish D. M., Valone F. H., Memoli V. A., Bzik D. J., Yao S. L. (1994). Soluble normal and mutated DNA sequences from single-copy genes in human blood. *Cancer Epidemiology, Biomarkers & Prevention*.

[15] Newman A. M., Bratman S. V., To J. (2014). An ultrasensitive method for quantitating circulating tumor DNA with broad patient coverage. *Nature Medicine*.

[16] Diaz L. A., Bardelli A. (2014). Liquid biopsies: genotyping circulating tumor DNA. *Journal of Clinical Oncology*.

[17] Romero D. (2015). Tracking ctDNA to evaluate relapse risk. *Nature Reviews. Clinical Oncology*.

[18] Bidard F. C., Madic J., Mariani P. (2014). Detection rate and prognostic value of circulating tumor cells and circulating tumor DNA in metastatic uveal melanoma. *International Journal of Cancer*.

[19] Martignetti J. A., Camacho-Vanegas O., Priedigkeit N. (2014). Personalized ovarian cancer disease surveillance and detection of candidate therapeutic drug target in circulating tumor DNA. *Neoplasia*.

[20] Goéré D., Daveau C., Elias D. (2008). The differential response to chemotherapy of ovarian metastases From colorectal carcinoma. *European Journal of Surgical Oncology*.

[21] Ganesh K., Shah R. H., Vakiani E. (2017). Clinical and genetic determinants of ovarian metastases from colorectal cancer. *Cancer*.

[22] Kemps P. G., Bol M., Steller E. J. A. (2021). Colon carcinoma presenting as ovarian metastasis. *Radiology Case Reports*.

[23] Cervantes A., Adam R., Roselló S. (2023). Metastatic colorectal cancer: ESMO Clinical Practice Guideline for diagnosis, treatment and follow-up. *Annals of Oncology*.

[24] Zulfiqar M., Koen J., Nougaret S. (2020). Krukenberg tumors: update on imaging and clinical features. *AJR. American Journal of Roentgenology*.

[25] Var T., Tonguc E. A., Ugur M., Altinbas S., Tokmak A. (2012). Tumor markers panel and tumor size of ovarian dermoid tumors in reproductive age. *Bratislavské Lekárske Listy*.

[26] Kotani D., Oki E., Nakamura Y. (2023). Molecular residual disease and efficacy of adjuvant chemotherapy in patients with colorectal cancer. *Nature Medicine*.

[27] Shah P. K., Aushev V. N., Ensor J. (2025). Circulating tumor DNA for detection of molecular residual disease (MRD) in patients (pts) with stage II/III colorectal cancer (CRC): final analysis of the BESPOKE CRC sub-cohort. *Journal of Clinical Oncology*.

[28] National Comprehensive Cancer Network Colon cancer (Version 5.2024). https://www.nccn.org/professionals/physician_gls/pdf/colon.pdf.

